# Overview of the biochemical and genetic processes in malignant mesothelioma*

**DOI:** 10.1590/S1806-37132014000400012

**Published:** 2014

**Authors:** Leonardo Vinícius Monteiro de Assis, Mauro César Isoldi

**Affiliations:** Institute of Biosciences, University of São Paulo, São Paulo, Brazil; Federal University of Ouro Preto, Ouro Preto, Brazil

**Keywords:** Occupational diseases, Mesothelioma, Genes, tumor suppressor, Oncogenes, Signal transduction

## Abstract

Malignant mesothelioma (MM) is a highly aggressive form of cancer, has a long latency period, and is resistant to chemotherapy. It is extremely fatal, with a mean survival of less than one year. The development of MM is strongly correlated with exposure to asbestos and erionite, as well as to simian virus 40. Although various countries have banned the use of asbestos, MM has proven to be difficult to control and there appears to be a trend toward an increase in its incidence in the years to come. In Brazil, MM has not been widely studied from a genetic or biochemical standpoint. In addition, there have been few epidemiological studies of the disease, and the profile of its incidence has yet to be well established in the Brazilian population. The objective of this study was to review the literature regarding the processes of malignant transformation, as well as the respective mechanisms of tumorigenesis, in MM.

## Introduction

Malignant mesothelioma (MM) is a rapidly growing cancer that results from unregulated proliferation of the mesothelial cells lining the pleural, peritoneal, and pericardial cavities. MM is typically but not exclusively related to exposure to mineral fibers, particularly asbestos and erionite.^(^ 1 ^)^ The latency period of MM, i.e., the time elapsed from exposure to the offending agent (in particular, the aforementioned mineral fibers) to diagnosis is long; however, the time elapsed from the onset of malignancy to diagnosis is indeed short, MM producing symptoms shortly after its initial growth.^(2)^

Histologically, MM can be classified as epithelial, biphasic, or sarcomatoid, the mean survival time being 18 months, 11 months, and 8 months, respectively.^(^ 3 ^)^ Malignant pleural mesothelioma (MPM) is the most common type of MM, accounting for approximately 70% of cases.^(^ 1 ^)^ As is the case with most MMs, MPM is commonly diagnosed at advanced stages, the survival rates for MPM being lower than 12 months.^(^ 4 ^)^ Malignant peritoneal mesothelioma is less common than MPM and accounts for approximately 30% of all MMs, being extremely aggressive (mean survival rate, 6-12 months).^(^ 5 ^,^ 6 ^)^

In addition to the fact that MM is highly resistant to chemotherapy and radiation therapy, the benefits of surgical removal are few, and there is controversy regarding the efficacy of surgical removal alone; furthermore, not all patients can undergo surgical removal.^(^ 7 ^)^ Apparently, improvements in survival rates have been achieved with a combination of surgical removal, chemotherapy, and radiation therapy; however, controversy remains regarding the efficacy and benefits of this practice.^(^ 6 ^,^ 8 ^,^ 10 ^)^ It is known that cisplatin is the most active drug in the treatment of MM, the use of cisplatin in combination with pemetrexed having been approved by the US Food and Drug Administration as a standard treatment for MM.^(^ 11 ^)^ However, various in vitro and in vivo studies have used other drugs, some of which have shown promising results.^(^ 12 ^)^

The first study to demonstrate that there was a relationship between asbestos and the development of MPM was conducted in South Africa in the 1960s.^(^ 13 ^)^ Since then, several studies have shown strong evidence that asbestos, especially amphibole asbestos, is associated with the development of MM.^(^ 2 ^,^ 7 ^)^ However, it is widely debated whether chrysotile asbestos is a human carcinogen. 

The biochemical mechanisms responsible for the genesis of MM as a result of asbestos exposure have yet to be fully understood. In broad terms, asbestos particles become trapped in lung tissue, generating a strong inflammatory response, with the participation of TNF-α and nuclear factor kappa B (NF-κB), which generate resistance to apoptosis and accumulation of DNA damage.^(^ 14 ^)^ The involvement of high mobility group protein B1, which is known to be an inflammatory marker, has recently been demonstrated. This protein increases the release of TNF-α and IL-1β, as well as increasing the activity of NF-κB.^(^ 15 ^)^ In addition to eliciting this inflammatory response, asbestos can generate reactive oxygen and nitrogen species, which lead to DNA structure damage and induce genotoxicity, thus favoring the development of MM.^(^ 16 ^,^ 17 ^)^

In the scientific literature, there is considerable debate regarding the role of chrysotile asbestos in the genesis of MM; there are reports that chrysotile asbestos cannot cause MM in humans. Although there is no doubt about the role of amphibole asbestos in the genesis of MM, there is still much debate regarding the role of chrysotile asbestos in MM, which is why chrysotile asbestos is still used in several countries, including Brazil. Here, we will not discuss this controversial issue, the nature of which is more political than scientific. However, the dangers of chrysotile asbestos cannot be ignored, which is why chrysotile asbestos and other types of asbestos are classified as human carcinogens, their use being considered unsafe regardless of the level of exposure.^(^ 18 ^)^ In addition to asbestos exposure, risk factors for the development of MM include erionite exposure, simian virus 40, and germline *BAP1* mutations.^(^ 2 ^,^ 19 ^,^ 21 ^)^

In several countries, the incidence of MM has increased significantly in recent years. Data from the USA show that the mean incidence of MM is 2,586 cases per year, with a cumulative total of 23,277 MM cases between 1999 and 2007, the incidence of MM in males being four times higher than that in females.^(^ 22 ^)^ In Brazil, there have been very few epidemiological studies, all of which were based on reported cases and on data from Brazilian National Ministry of Health databases. This makes it difficult to provide a realistic picture of MM in the country. 

Despite the aforementioned difficulties, one group of Brazilian researchers conducted a study^(^ 23 ^)^ in which the Brazilian National Mortality Database was used in order to estimate the incidence of MM in Brazil. Between 1980 and 1995, the authors of that study used the International Classification of Diseases, 9th revision (ICD-9), codes 163.0, 163.1, 163.8, and 163.9, and all cases of pleural neoplasm were considered to be cases of mesothelioma. Between 1996 and 2003, the authors used the International Classification of Diseases, 10th revision (ICD-10), codes C45.0, C45.1, C45.2, C45.7, C45.8, C45.9, and C38.4. That study showed that the mortality rate for MM was 0.56 per 1,000,000 population in 1980, having increased by 55% in 2003.^(^ 23 ^)^ In addition, the study showed that the male-to-female ratio of patients with MM was nearly 1:1,^(^ 23 ^)^ which is quite different from the 5:1 ratio found in a recent study conducted in the UK.^(^ 24 ^)^ However, given the methodological limitations of the aforementioned study,^(^ 23 ^)^ it is possible that its findings do not reflect the true incidence of MM in Brazil. The limitations of the aforementioned study^(^ 23 ^)^ include the low quality of the data from the Brazilian National Mortality Database, the underreporting of cases in some Brazilian states, the use of two different revisions of the ICD (i.e., ICD-9 and ICD-10), and the fact that, according to ICD-9, all pleural neoplasms are MMs. Therefore, the findings of that study are difficult to interpret, and the real incidence of MM in Brazil remains unknown. However, despite the aforementioned methodological difficulties,^(^ 23 ^)^ the information provided by that study is extremely useful for estimating the incidence of MM, as well as reinforcing the idea that the appropriate authorities should monitor the incidence of MM more closely in order to provide reliable data, as is done in the USA.^(^ 22 ^)^ In addition to the abovementioned epidemiological study,^(^ 23 ^)^ studies have been conducted in Brazil in an attempt to improve the diagnosis of MM^(^ 25 ^,^ 26 ^)^ and find prognostic markers of MM.^(^ 27 ^)^

Given its aggressiveness and increasing incidence worldwide, MM and its main etiologic agent (i.e., asbestos) have been the subject of international discussions aimed at banning the trade of asbestos worldwide. In Brazil, asbestos is regulated by Law no. 9,055; all forms of asbestos are prohibited, with the exception of chrysotile.^(^ 28 ^)^ There have been few studies investigating MM and the profile of individuals diagnosed with MM in Brazil,^(^ 26 ^,^ 29 ^,^ 33 ^)^ further studies being therefore required. 

The primary objective of the present review was to provide an overview of how MM uses the cellular machinery in order to promote its growth, i.e., an overview of the genes and pathways that are activated or deactivated. This knowledge is important for the development of new drugs and therapies for this aggressive cancer. We did not seek to review the roles of asbestos and other environmental exposure factors in the development of MM. Our primary objective was to review the principal biochemical and genetic events occurring in MM and their consequences in the process of malignant transformation, in an attempt to strengthen the Portuguese-language scientific literature, which lacks a review of studies on this topic.

## Genes and biochemical pathways involved in MM

An understanding of the cellular processes that favor or assist in the process of MM development is of utmost importance for the creation of therapies aimed at activating or deactivating certain biochemical pathways, the principal effect being tumor growth suppression. Research groups worldwide have been working on this, and there have been major advances, which have aided in the treatment of MM. Below, we briefly describe the genes that play a key role in the development of MM. For a more detailed analysis of the mechanics of the genes involved in MM, please refer to recently published review articles by our research group.^(^ 21 ^,^ 34 ^)^

**Figure 1:**
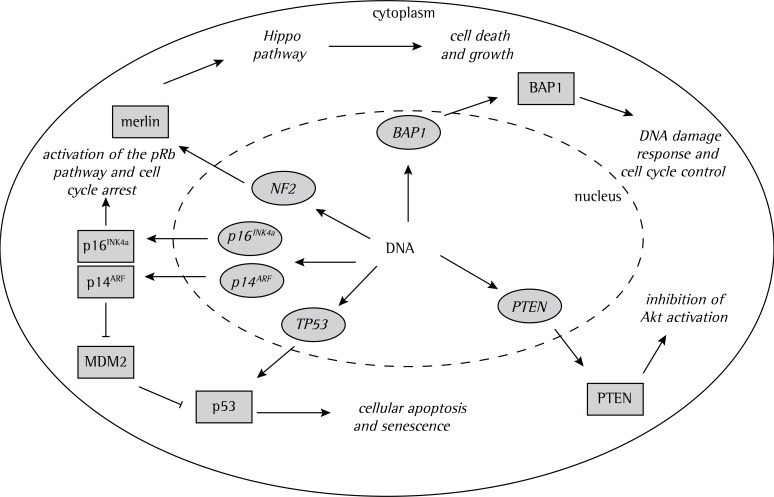
Genes and proteins involved in the development of malignant mesothelioma. The p16INK4a protein activates the retinoblastoma protein (pRb) pathway, and the p14ARF protein modulates p53. The NF2 gene encodes the merlin protein, which acts as an upstream regulator of the Hippo pathway. The BAP1 gene encodes BRCA1 associated protein-1, which plays a role in DNA damage response and cell cycle control. The PTEN gene encodes the PTEN protein, which is an important negative regulator of the PI3K/Akt pathway. The p53 protein plays a key role in apoptosis control and cellular senescence.

It is known that each type of cancer uses a certain "group" of genes in order to grow; however, the group of genes used depends on cancer type and stage. Certain patterns of gene activation and deactivation occur in all types of cancer and are explored in the development of drugs and therapies. In the particular case of MM, the genes whose roles are well established are *p16* *INK4a*, *p14* *ARF*, *NF2*, and *BAP1*. Although the roles of the *TP53* and *PTEN* genes are well established in various types of cancer, their roles in MM remain controversial. Figure 1 shows an overview of the roles of the aforementioned genes in MM. 

### p16INK4a and p14ARF

Located on chromosome 9p21, the *p16* *INK4a *and *p14* *ARF* genes are important tumor growth suppressors and encode two distinct proteins, namely p16^INK4a^ and p14^ARF^. The p16^INK4a^ protein is a cyclin-dependent kinase inhibitor and plays a role in the hyperphosphorylation of the retinoblastoma protein. This results in inactivation of the retinoblastoma protein and, consequently, failure of cell cycle arrest. In contrast, the p14^ARF^ protein inhibits the degradation of p53 through its interaction with murine double minute 2 protein (MDM2).^(^ 35 ^)^ The loss of these vicinal genes has a major impact on cell cycle control, and it is therefore possible to infer the reason why these are the most frequently mutated genes in MM. 

The literature shows that *p16* *INK4a* and *p14* *ARF* are deleted in 80-90% of cases of MM.^(^ 36 ^,^ 37 ^)^ Approximately 70% of all cases of epithelial MM and nearly 100% of all cases of biphasic or sarcomatoid MM show changes in *p16* *INK4a* and *p14* *ARF*.^(^ 38 ^)^ The literature shows that *p16* *INK4a* and *p14* *ARF*, as well as their respective proteins, play important roles in cell cycle control and that their inactivation is most frequently involved in the malignant transformation of MM.

### NF2 

Located on chromosome 22q12, the *NF2* gene encodes a protein designated merlin, which has a sequence of 595 amino acids and plays an important role in the upstream regulation of the cascade of the Hippo pathway, which will be explained later. In the mid-1990s, inactivation of the *NF2* gene was reported in approximately 40% of all cases of MM.^(^ 39 ^)^ Subsequent studies have demonstrated the importance of *NF2* inactivation in MM.^(^ 40 ^)^ Although *NF2* mutations have been found in 38% of cases of MPM, an absence of *NF2* mutations has recently been reported in non-small cell lung cancer, this being a possible approach to the differential diagnosis of the two.^(^ 41 ^)^ Therefore, mutations/alterations in the *NF2* gene are important to the development of MM and currently constitute the second most common alteration in MM. 

### BAP1

The *BAP1* gene is a tumor suppressor gene that is located on chromosome 3p21.3 and encodes the protein BAP1, which plays an important role in the ubiquitin-proteasome pathway in histone deubiquitination, regulation of cell cycle progression, modulation of chromatin, gene transcription, and DNA repair.^(^ 42 ^)^

Germline *BAP1* mutations have recently been detected in families with a high incidence of MM, characterizing a syndrome that predisposes to MM, uveal melanoma, and, possibly, other cancers.^(^ 19 ^,^ 42 ^,^ 43 ^)^ In addition to germline mutations, somatic mutations have been identified in approximately 20% of all cases of MM.^(^ 44 ^,^ 45 ^)^ Studies^(^ 19 ^,^ 42 ^,^ 43 ^)^ of the effects of germline mutations on cancer development have provided a major breakthrough, given that cancer is often associated with the effects of somatic mutations related or unrelated to external factors, including exposure to asbestos, radiation, and cigarette smoke. Therefore, it is of paramount importance to gain a better understanding of the genes involved in the development of MM, as well as of the mechanisms by which germline mutations contribute to the development of MM, because individuals with such genetic susceptibilities should avoid exposure to risk factors. To that end, there is a need for techniques that can detect such mutations in the population in an inexpensive and reproducible manner, given that such screening is currently performed on a small scale and in scientific studies. In individuals suspected of having *BAP1* cancer syndrome, early diagnosis is essential to prevent the onset of diseases associated with *BAP1* mutations. Therefore, a multidisciplinary approach involving family physicians, pathologists, and geneticists is required in order to diagnose, monitor, advise, and treat individuals and families with this syndrome. There is a need for knowledge and training of health professionals (especially physicians) regarding the clinical signs of *BAP1* cancer syndrome, which can result in catastrophic harm to patients if it is not diagnosed early.^(^ 42 ^)^

### TP53

Known as a DNA guardian, the p53 protein is encoded by the *TP53* gene. The p53 protein plays a role in various cellular functions that are critical for well-orchestrated cell control. In addition, *TP53* mutations are found in approximately 50% of all cancer cases, and, in most other cases, *TP53* is inactivated by mutations in other genes, by viral proteins, or both.^(^ 46 ^,^ 47 ^)^

From a mechanistic standpoint, several research groups have focused on gaining a better understanding of the various mechanisms of p53 cell function control. The first clues as to the role of p53 in mediating apoptosis were provided by a study published in the 1990s.^(^ 48 ^)^ A new mechanism of action of p53 has recently been identified and is believed to be one of the main mechanisms used to fight the process of malignant transformation, i.e., induced cellular senescence.^(^ 49 ^)^ However, the role of the p53 protein in MM has yet to be well defined; intriguingly, in most cases, MM does not neutralize p53 activity in a direct manner, i.e., through *TP53* mutations. Studies have shown that the *TP53* gene is present in its natural state, i.e., without mutations.^(^ 50 ^,^ 51 ^)^ However, the absence of mutations in a given gene does not necessarily imply that the gene is functioning normally, given that there are various gene regulation mechanisms that can cause gene inactivation, including DNA methylation (epigenetic regulation) and RNA interference (post-transcriptional regulation).^(^ 52 ^)^

It can be speculated that other gene mutations (such as the aforementioned mutations) can lead to malignant transformation in MM, resulting in reduced selective pressure for *TP53* inactivation. In addition, it is plausible to assume that the malignant transformation of MM occurs through pathways that are independent of p53 activity. The mechanisms leading to the maintenance of wild-type *TP53* in MM have yet to be fully understood. Mutations/alterations in the *TP53* gene do not seem to be critical to the development and progression of MM.^(^ 53 ^,^ 54 ^)^

### PTEN

Discovered in 1997 by two independent research groups,^(^ 55 ^,^ 56 ^)^ the *PTEN* gene is a common deletion on chromosome 10. Monoallelic mutations are common in various types of cancer; however, homozygous *PTEN* mutations are frequently found in advanced cancers, such as endometrial cancer and glioblastoma.^(^ 57 ^)^ Interestingly, *PTEN* is heavily regulated by various gene regulation processes, such as RNA interference, methylation, acetylation, oxidation, and ubiquitination. Therefore, analysis of gene status (i.e., mutation levels) is important but should not be used as the only predictor of gene activation and function. Analysis of protein expression levels is also required, given the potential association between protein expression and susceptibility to cancer development, as is the case with *PTEN*.^(^ 52 ^,^ 58 ^)^

The activity of PTEN results from the ability to antagonize the signaling pathway of the phosphatidylinositol 3-kinase (PI3K) pathway through the dephosphorylation of phosphatidylinositol-3,4,5-trisphosphate (PIP3) to phosphatidylinositol 4,5-bisphosphate (PIP2). It is known that PIP3 is a second messenger responsible for the activation of Akt (i.e., PKB), which in turn sends signals necessary for cell growth, survival, and proliferation. In fact, various types of cancer display overexpression in this biochemical pathway. This results in uncontrolled cell growth. Loss of PTEN activity results in accumulation of PIP3 and, consequently, overactivation of Akt, PTEN being therefore commonly used in malignant processes.^(^ 59 ^)^ In addition to having cytoplasmic activities, PTEN has nuclear activities that are important for cell cycle control and genomic stability.^(^ 59 ^,^ 60 ^)^

**Figure 2:**
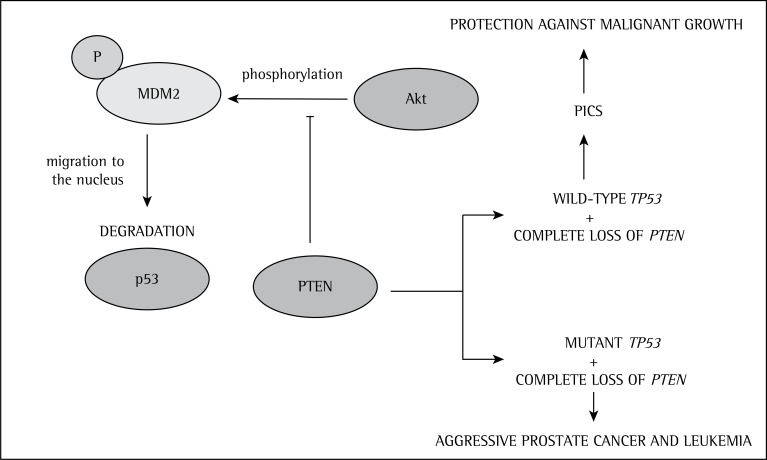
The PTEN protein protects p53 from degradation by inhibiting the migration of murine double minute 2 protein (MDM2) to the nucleus. There is a cross-talk mechanism between PTEN and p53. The association between complete loss of PTEN and a wild-type TP53 results in a senescence mechanism designated PTEN loss-induced cellular senescence (PICS), which is an important mechanism against malignant growth. The loss of PTEN in a cellular context with a mutant TP53 results in prostate cancer that is more aggressive and leukemia, both mechanisms having been demonstrated in animals.

Interestingly, PTEN can regulate p53 levels independently of its activity as a phosphatase by maintaining p53 acetylation (Figure 2).^(^ 61 ^)^ In addition, PTEN inhibits MDM2 phosphorylation, which is required for nuclear migration and, consequently, p53 degradation. Therefore, PTEN can protect p53 from the degradation of MDM2.^(^ 62 ^,^ 63 ^)^

A new mechanism has recently been identified in prostate cancer, a mechanism in which complete loss of *PTEN* in combination with wild-type *TP53* surprisingly induced a strong cellular senescence response, which resulted in the inhibition of malignant cell growth. However, the combination of complete loss of *PTEN* and wild-type *TP53* was associated with a more severe form of prostate cancer. Therefore, it is plausible to speculate the reason why complete (homozygous) loss of *PTEN* is restricted to advanced cancers.^(^ 52 ^)^

The role of *PTEN* in MM remains controversial, given that the PI3K/Akt pathway is known to be overexpressed; however, whether this overexpression is due to the absence of *PTEN* or to *PTEN* inactivation and the role of *PTEN* in the development of MM are still a matter of debate.^(^ 64 ^-^ 67 ^)^

### DNA methylation and microRNA

Recent studies have shown that DNA methylation and microRNA expression play an important role in cancer development and should be explored in the diagnosis and treatment of cancer. In MM, epigenetic analysis of the methylation profile of several genes allowed the distinction between normal and malignant tissues, a fact that is of great importance because of the difficulty in distinguishing normal and malignant tissues^(^ 68 ^)^ and because epigenetic analysis of the methylation profile can be a powerful tool in the diagnosis of MM.^(^ 69 ^)^

MicroRNA studies have shown interesting results. MicroRNAs can regulate and modulate gene expression, microRNA expression being severely altered in cancer.^(^ 70 ^)^ MicroRNA expression in normal tissue has been shown to be different from microRNA expression in malignant tissue, and specific microRNA expression profiles have been found in each histological type of MM.^(^ 71 ^)^ Studies have proposed the use of microRNA as a diagnostic tool,^(^ 71 ^)^ a prognostic marker,^(^ 72 ^)^ and a treatment option for MM.^(^ 73 ^)^ Therefore, the future looks promising for these two parameters and their potential benefits in the diagnosis and treatment of MM. 

## Biochemical pathways involved in the development of MM^94^ ^95^ ^96^ ^97^ ^98^ ^99^ ^100^

**Figure 3:**
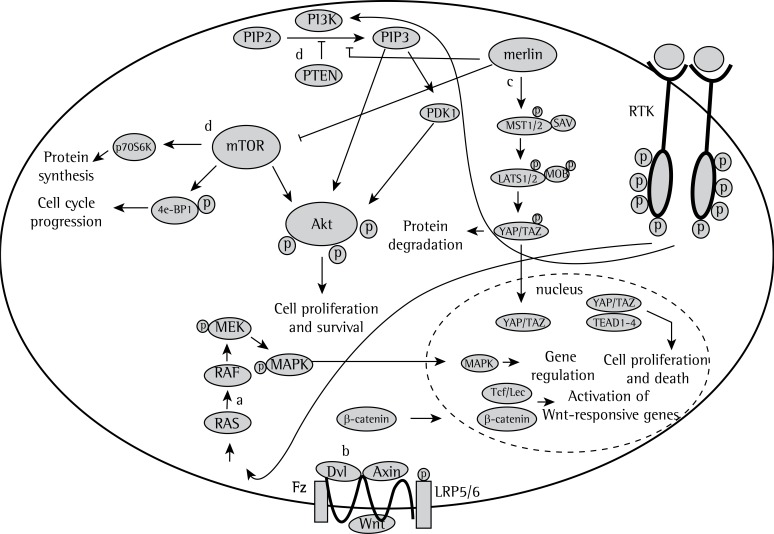
Biochemical pathways most commonly altered in malignant mesothelioma. In a, receptor tyrosine kinases are frequently activated in malignant mesothelioma, thus increasing the Ras and PI3K pathways. The Ras pathway activates the Raf pathway, which phosphorylates mitogen-activated protein kinase kinase (MEK). In turn, MEK phosphorylates mitogen-activated protein kinase (MAPK), which migrates to the nucleus, thus regulating gene expression. In b, the Wnt pathway controls various cellular processes. In the presence of a Wnt ligand, a complex involving disheveled homolog (Dvl), Axin, frizzled (Fz), and low-density lipoprotein receptor-related proteins (LRP5/6), it leads to inhibition of ß-catenin phosphorylation and degradation. Consequently, ß-catenin migrates to the nucleus, where it interacts with the Tcf/Lef complex, thus leading to the activation of Wnt-responsive genes. In c, the merlin protein is encoded by the NF2 gene and inhibits the PI3K pathway and mTOR, acting as an upstream regulator of the Hippo pathway. A biochemical cascade is initiated by a stimulus, macrophage stimulating 1/2 (MST1/2) phosphorylating salvador homolog 1 (SAV1), large tumor suppressor 1/2 (LATS1/2), and Mps one binder kinase 1 (MOB1). The MST1/2 and SAV1 complex phosphorylates LATS1/2; the LATS1/2 and MOB1 complex interacts directly and phosphorylates YAP/TAZ. Phosphorylated YAP/TAZ leads to protein degradation, whereas dephosphorylated YAP/TAZ enters the nucleus and binds to TEAD1-4 transcription factors in order to regulate genes involved in cell proliferation and death. In d, the PI3K/Akt/mTOR pathway is activated by the conversion of phosphatidylinositol-3,4,5- trisphosphate (PIP3) to phosphatidylinositol 4,5-bisphosphate (PIP2), PTEN acting as an antagonist of this activation. PIP3, pyruvate dehydrogenase kinase, isozyme 1 (PDK1), and mammalian target of rapamycin (mTOR) phosphorylate protein kinase B (Akt). Activated Akt participates in processes that are central to cell proliferation, survival, and motility.

Below, we briefly describe the biochemical pathways most commonly used in the malignant transformation of MM. These pathways are summarized in Figure 3. For a more detailed analysis of the mechanics of the pathways involved in MM, please refer to recently published review articles by our research group.^(^ 21 ^,^ 34 ^)^

### Receptor tyrosine kinases 

Receptor tyrosine kinases constitute a large family of receptors that regulate the cell cycle and are often activated in MM.^(^ 74 ^)^ Among receptor tyrosine kinases, EGFR was detected in 44% of all cases of MPM.^(^ 75 ^)^ In addition, VEGF is expressed in MM and is associated with decreased patient survival.^(^ 76 ^)^ In addition, insulin-like growth factor and its receptor are also active in MM.^(^ 77 ^)^

Activation of these receptors results in the activation of biochemical cascades that lead to the transduction of abnormal cell growth signals, principally through the Ras/MAPK pathway^(^ 78 ^,^ 79 ^)^ and the PI3K/Akt pathway^(^ 80 ^)^ in MM. 

### PI3K/Akt/mTOR

The PI3K pathway regulates various processes that are vital to cells, including survival, metabolism, and proliferation. A major product of the PI3K pathway, PIP3 acts as a second messenger essential for the translocation of Akt to the plasma membrane, where Akt is phosphorylated. Phosphorylated Akt is responsible for sending biochemical signals responsible for cell proliferation and resistance to apoptosis.^(^ 64 ^)^ The *PIK3CA* gene encodes the catalytic unit p110α, which is known for its ability to activate the PI3K pathway by converting PIP2 to PIP3.^(^ 81 ^)^ It is known that Akt is phosphorylated by mammalian target of rapamycin (mTOR), and the mTOR complex plays an important role in energy balance and growth, being therefore a therapeutic target of interest in patients with MM.^(^ 82 ^)^

In MM, the PI3K/Akt/mTOR pathway is overexpressed,^(^ 64 ^,^ 80 ^,^ 83 ^)^ inhibition of the activity of certain pathway components, such as PI3K and mTOR, being therefore an excellent therapeutic pathway.^(^ 84 ^)^ However, the applicability of such drugs in clinical practice has proved frustrating.^(^ 85 ^)^

Recently, in an elegant study,^(^ 86 ^)^ it was demonstrated that activation of colony-stimulating factor 1 receptor (CSF1R) can generate clonogenicity and resistance in untransformed mesothelial cells. It was also demonstrated that, in primary MPM cultures and MPM cell lines, there are subpopulations of cells expressing CSF1R, which is responsible for resistance to pemetrexed via Akt and β-catenin signaling. Another interesting finding of that study was that the abovementioned subset of cells accounts for less than 10% of the total number of cells in culture, this small proportion being responsible for resistance to pemetrexed in cell lines and primary cultures; therefore, CSF1R plays an important role in the survival of cells that do not express it.^(^ 86 ^)^ Given that CSF1R greatly influences cell survival and that CSF1R expression is higher in MM than in normal tissue, pharmacological inhibition of CSF1R in humans is an attractive and promising strategy to overcome the high resistance to chemotherapy observed in MM patients and expand the limited therapeutic armamentarium currently available to combat MM.^(^ 86 ^)^

### Ras/MAPK

The Ras/MAPK pathway consists of several components, such as surface receptors and transcription factors, which regulate gene expression. The Ras/MAPK pathway is one of the most frequently deregulated pathways in human cancer and controls vital cellular processes, such as proliferation, growth, and senescence, as well as regulating apoptosis through its interaction with various members of the B-cell lymphoma (Bcl) family of proteins.^(^ 87 ^)^ The major components of the Ras/MAPK pathway are Ras, Raf, MEK, and MAPK, which are susceptible to mutations/alterations and therefore favor the process of malignant transformation. Given the importance of the Ras/MAPK pathway, several drugs have been developed, some of which are under clinical trial.^(^ 88 ^)^

Studies have shown higher expression of MAPK in MM than in normal lung tissue,^(^ 89 ^)^ as well as prolonged MAPK activation after exposure to asbestos.^(^ 78 ^)^ This shows that the Ras/MAPK pathway plays a role in MM growth and that its inhibition can yield interesting results in the treatment of MM. 

### The Bcl family of proteins and apoptosis

Responsible for the control of apoptosis, the Bcl family of proteins is divided into two major classes, namely pro-apoptotic proteins and anti-apoptotic proteins.^(^ 90 ^)^ Like other types of cancer, MM is resistant to apoptosis, and this hinders the destruction of malignant cells by traditional chemotherapy.^(^ 91 ^)^ Various anti-apoptotic members of the Bcl family of proteins are expressed by MM,^(^ 92 ^)^ and this reduces the efficacy of traditional chemotherapy. Bcl family inhibitors have been developed and have shown interesting results; however, the clinical application of such drugs remains unknown.^(^ 93 ^)^

### Hippo

The Hippo pathway controls cell proliferation, growth, and death via a complex cascade of biochemical events that result in gene regulation. (94) In MM cells, the Hippo pathway was identified

by the loss of LATS2 and YAP expression, as well as by inactivation of an upstream regulator of the pathway, such as merlin, which is encoded by the NF2 gene.(95) The Hippo pathway is used in the malignant transformation of MM. Further studies are needed in order to understand the mechanisms by which MM uses the Hippo pathway for its benefit.

### Wnt

The Wnt pathway regulates important cellular processes, such as cell proliferation, polarity, and death during embryonic development and in the process of tumor progression.(96) In broad terms, activation of the Wnt pathway can be canonical (i.e., a change in the transcription process) or non-canonical (i.e., activation of non-transcriptional processes).

β-catenin is the principal Wnt pathway transcriptional effector, acting in the nucleus and forming a molecular complex that leads to the activation of specific genes.(97) The Wnt pathway has been shown to be altered in MM(98,99) and has been implicated in decreased patient survival.(100)

## Relevance of altered signaling processes in cancer

An understanding of the complex and enigmatic biochemical and molecular processes that occur during malignant transformation is of paramount importance for the development of new drugs and therapies. The search for an understanding of how malignant cells can subvert the cellular machinery and all cell cycle control systems has been exhaustive, resulting in new drugs and therapies that increase the chances of survival of patients with MM. 

### Individualization: the future of cancer treatment

A deep understanding of how cancer uses the cellular machinery to drive its growth is of paramount importance; each type of cancer uses distinct genes and pathways, thus generating "patterns" of activation and inactivation.^(^ 101 ^)^ These "patterns" can provide important clues for the development of cancer-specific drugs and therapies. 

A deep understanding of the molecular processes occurring in a given patient is within the scope of personalized medicine, the objective of which is to treat each disease (e.g., cancer) individually (because of the large variability in physiological processes) in an attempt to improve treatment and prognosis. Although this approach is still in its infancy, it might be used in clinical practice in the future.^(^ 102 ^)^

In patients receiving certain drugs that are metabolized by specific enzymes, enzyme profile analysis is sometimes recommended. Genetic variations in these enzymes culminate in changes in the pharmacokinetic and pharmacodynamic profiles of drugs, resulting in increased adverse effects and treatment failure.^(^ 103 ^)^

Although a "one-size-fits-all" approach has been used in the treatment of cancer, an individualized approach is required. In order to choose the best drug or drugs for individual patients (i.e., specific drugs for cancer targets), it is essential to know the receptors and pathways expressed by a given cancer type. This individualized approach improves treatment and increases the chances of survival. Advances in knowledge and technology regarding the process of malignant transformation have resulted in the development of personalized medicine, which is based on the analysis of deregulated cellular processes in individual patients and the use of specific therapeutic tools, therefore increasing the chances of successful treatment.

## Final thoughts and future directions

The primary motivation for the present study was the lack of studies reviewing the Portuguese-language literature on genetic and biochemical processes in MM. In fact, MM is not widely studied in Brazil; there are few research groups dedicated to the epidemiological study of MM in the country. In addition, there is a lack of reliable data on the profile, incidence, and prevalence of MM in the Brazilian population. 

Our research group has been engaged in gaining a better understanding of the cell signaling processes that contribute to the tumorigenesis of MM. The primary objective of the present study was to provide an overview of the most common genetic and biochemical events in the malignant transformation of MM. As previously mentioned, we did not seek to provide an extensive review of the pathways involved in MM; our objective was to provide an overview of cell signaling processes in MM. We conclude that MM is a highly aggressive cancer and has a long latency period, with very low survival rates. Worldwide, great efforts have been made to gain a better understanding of the process of tumorigenesis in MM and propose and develop alternatives for the treatment of this aggressive cancer. It should be emphasized that it was outside the scope of the present study to analyze factors associated with MM, such as exposure to asbestos, erionite, and simian virus 40. 

Mutations are common in MM, affecting genes such as *p16* *INK4a*, *p14* *ARF*, *NF2*, and *BAP1*, whose mutations are commonly somatic. In addition, germline *BAP1* mutations have recently been identified, conferring susceptibility to the development of MM and other types of cancer. Although *TP53* and *PTEN* are known to play major roles in other types of cancer, their roles in MM require further investigation. In addition to the aforementioned genes, the PI3K/Akt/mTOR, Ras/MAPK, Bcl, Hippo, and Wnt pathways and their components are the most altered pathways in MM. Several studies have focused on the modulation of these pathways for a safe and effective reduction in the malignant growth of MM, including in vitro studies, animal studies, and clinical trials. 

It is clear that further studies are needed in order to improve the understanding and characterization of the genes and pathways involved in the tumorigenesis of MM, given that few studies in Brazil have addressed this issue. In addition, there is a need for epidemiological studies aimed at analyzing the incidence of MM in the Brazilian population and establishing a realistic and reliable profile of MM in the country. 
