# ERRATUM

**DOI:** 10.1590/S1806-37132014000500019

**Published:** 2014

**Manuscript:** Overview of the biochemical and genetic processes in malignant mesothelioma

**Publication:**

**DOI:**10.1590/S1806-37132014000400012 

On page 429 of the original publication, in the third line of the abstract, where is written "The development of MM is strongly correlated with exposure to asbestos and erionite, as well as to simian virus 40." should be read "The development of MM is strongly correlated with exposure to asbestos and with other factors, such as erionite and simian virus 40."

