# Supplementary material for: Overview of the biochemical and genetic processes in malignant mesothelioma
Source: J Bras Pneumol. 2014 Jul-Aug;40(4):429–42. doi: 10.1590/S1806-37132014000400012 (PMC4201175; doi:10.1590/S1806-37132014000400012)
Supplement: Translation

# Translation: Panorama dos processos bioquímicos e genéticos presentes no mesotelioma maligno*

**Authors:** Leonardo Vinícius Monteiro de Assis, Mauro César Isoldi

**Affiliations:** Instituto de Biociências, Universidade de São Paulo, São Paulo (SP) Brasil; Universidade Federal de Ouro Preto, Ouro Preto (MG) Brasil

**Keywords:** Doenças profissionais, Mesotelioma, Genes supressores de tumor, Oncogenes, Transdução de sinal

## Abstract

O mesotelioma maligno (MM) é um câncer extremamente agressivo, com elevado período de latência e resistente aos protocolos de quimioterapia, além de ser extremamente fatal, com taxa de sobrevivência média inferior a um ano. O desenvolvimento do MM é fortemente correlacionado com a exposição ao amianto e erionita, assim como ao vírus símio 40. Apesar de vários países terem banido o uso de amianto, o MM tem se mostrado de difícil controle e sua incidência tende a aumentar nos próximos anos. No Brasil, o MM não é amplamente estudado do ponto de vista genético e bioquímico. Além disso, poucos estudos epidemiológicos foram realizados até o momento, e o perfil de incidência do MM não está bem estabelecido na população brasileira. O objetivo deste estudo foi revisar a literatura em relação ao processo de transformação maligna e seus respectivos mecanismos de tumorigênese no MM.

## Introdução

O mesotelioma maligno (MM) é um câncer de crescimento rápido que resulta da proliferação desregulada das células mesoteliais que revestem as cavidades pleurais, peritoneais e pericárdicas. O MM está tipicamente relacionado, mas não exclusivamente, com a exposição às fibras de minerais, em especial, de amianto e erionita.^(^ 1 ^)^ Seu período de latência, ou seja, o período entre a exposição ao agente agressor, em particular às fibras minerais citadas acima, até o diagnóstico é longo; entretanto, o tempo entre o crescimento inicial maligno até o diagnóstico é de fato rápido, produzindo sintomas em um curto intervalo de tempo.^(^ 2 ^)^

No que tange a sua divisão histológica, o MM é classificado em três categorias: epitelial, bifásica e sarcomatoide, as quais apresentam períodos de sobrevivência médios de 18, 11 e 8 meses, respectivamente.^(^ 3 ^)^ O mesotelioma pleural maligno (MPM) é o tipo mais comum de MM, correspondendo a aproximadamente 70% dos casos.^(^ 1 ^)^ Comumente diagnosticado em estágios avançados, como a grande maioria dos MMs, o MPM possui taxas de sobrevivências inferiores a 12 meses.^(^ 4 ^)^ Além do MPM, o mesotelioma peritoneal maligno é menos frequente e representa cerca de 30% de todos os MMs, sendo extremamente agressivo, com taxas de sobrevivência médias de 6 a 12 meses.^(^ 5 ^,^ 6 ^)^

O MM apresenta elevada resistência aos diversos protocolos de quimioterapia e radioterapia. A remoção cirúrgica apresenta poucos benefícios, sendo sua eficácia bastante controversa quando utilizada de forma única, além de não ser aplicável a todos os pacientes.^(^ 7 ^)^ Aparentemente, melhorias nas taxas de sobrevivência têm sido alcançadas com protocolos triplos, que envolvem a remoção cirúrgica associada a quimioterapia e radioterapia; contudo, ainda não há um consenso sobre a eficácia de tal prática, e seus benefícios, até o presente momento, são controversos.^(^ 6 ^,^ 8 ^-^ 10 ^)^ Sabe-se que a cisplatina é o fármaco mais ativo nesse câncer; logo, a associação desse fármaco com o pemetrexede foi aprovada pelo *Food and Drug Administration* para o tratamento padrão desse câncer.^(^ 11 ^)^ Contudo, diversos estudos têm utilizado vários fármacos, tanto in vitro como in vivo, que apresentam uma gama de diferentes respostas e resultados, sendo que alguns desses fármacos apresentam resultados promissores.^(^ 12 ^)^

O primeiro estudo que demonstrou a relação entre o amianto e o desenvolvimento de MPM ocorreu na África do Sul na década de 1960.^(^ 13 ^)^ Desde então, diversos estudos têm demonstrado fortes evidências de que o amianto, principalmente o tipo anfibólio, está de fato associado com o desenvolvimento de MM.^(^ 2 ^,^ 7 ^)^ Todavia, ainda há um grande debate mundial se o tipo crisotila seria carcinogênico em humanos. 

No que tange aos mecanismos bioquímicos responsáveis pela gênese do MM devido à exposição ao amianto, esses ainda não são muito bem entendidos. Contudo, em linhas gerais, sabe-se que partículas de amianto são aprisionadas no tecido pulmonar, gerando assim uma forte resposta inflamatória, com a participação de TNF-α e *nuclear factor kappa B* (NF-κB, fator nuclear kappa B), que geram resistência a apoptose e acúmulo de dano ao DNA.^(^ 14 ^)^ Mais recentemente, o envolvimento da *high-mobility group box 1 protein* (proteína de grupo de alta mobilidade B1), a qual é conhecida por ser um marcador inflamatório, foi demonstrado. Essa proteína aumenta a liberação de TNF-α e IL-1β, além de aumentar a atividade do NF-κB.^(^ 15 ^)^ Além dessa resposta inflamatória, o amianto é capaz de gerar espécies reativas de oxigênio e de nitrogênio, as quais levam a danos na estrutura do DNA, induzido assim a genotoxicidade e favorecendo o desenvolvimento do mesmo.^(^ 16 ^,^ 17 ^)^

Na literatura científica há um grande debate sobre a participação do amianto do tipo crisotila na gênese do MM, pois há relatos de que o amianto crisotila não é capaz de causar MM em humanos. Deve ser enfatizado que não restam dúvidas sobre o potencial do amianto do tipo anfibólio na gênese do MM; contudo, há ainda um grande debate sobre essa capacidade do amianto crisotila, argumento esse ainda utilizado por vários países, incluindo o Brasil, para a justificativa do uso desse tipo de amianto. No presente estudo, não iremos discutir essa questão tão controversa, a qual possui um cunho mais político do que científico, mas não podemos ignorar o perigo que as partículas de amianto crisotila apresentam, fato esse que levou a classificação do amianto crisotila e dos demais tipos de amianto como carcinogênicos em humanos, sem quantidades definidas como seguras para seu uso.^(^ 18 ^)^ Além do amianto, estudos recentes têm demonstrado outros fatores associados ao desenvolvimento do MM, tais como a exposição ao mineral erionita, o vírus símio 40 e a mutação germinativa no gene *BAP1*.^(^ 2 ^,^ 19 ^-^ 21 ^)^

A incidência de MM tem crescido significativamente nos últimos anos em diversos países. Dados dos EUA demonstram que a incidência do MM está em média de 2.586 casos por ano, com um total acumulado de 23.277 casos de MM entre 1999 e 2007, sendo que a incidência na população masculina é quatro vezes superior à feminina.^(^ 22 ^)^ No Brasil, há pouquíssimos estudos epidemiológicos, os quais se baseiam em notificações e nos bancos de dados do Ministério da Saúde, o que torna difícil o conhecimento de um quadro realístico da situação do MM em nosso país.

Mesmo com essas dificuldades, um estudo conduzido por um grupo brasileiro^(^ 23 ^)^ utilizou o Sistema de Informação sobre Mortalidade para estimar a incidência do MM no Brasil. Durante o período entre 1980 e 1995, foi utilizada a Classificação Internacional de Doenças, 9ª revisão (CID-9) e os códigos 163.0, 163.1, 163.8 e 163.9, além de considerar todos os casos de neoplasia pleural como mesotelioma. Entre os anos de 1996 e 2003, foi utilizada a 10ª revisão (CID-10) e os códigos C45.0, C45.1, C45.2, C45.7, C45.8, C45.9 e C38.4. Aquele estudo demonstrou que, em 1980, a taxa de mortalidade por MM era de 0,56 por milhão de habitantes, crescendo 55% em 2003.^(^ 23 ^)^ Além disso, foi demonstrado que a taxa de MM em homens e mulheres fora praticamente de 1:1,^(^ 23 ^)^ dados bem distintos de um recente estudo britânico, que encontrou uma proporção de 5:1 em homens e mulheres, respectivamente.^(24) ^Entretanto, aquele estudo^(^ 23 ^)^ apresenta algumas limitações metodológicas, e, portanto, suas conclusões podem não representar a verdadeira incidência do MM no Brasil. Entre as limitações daquele estudo^(^ 23 ^)^ estão a baixa qualidade dos dados do Sistema de Informação sobre Mortalidade, a baixa notificação em alguns estados brasileiros, a utilização de duas classificações CID e a classificação de todas as neoplasias pleurais como MM na CID-9. Logo, tendo em vista essas limitações, as conclusões daquele estudo são de difícil interpretação, abrindo-se então questionamentos sobre a real incidência do MM no Brasil. No entanto, mesmo com tais dificuldades metodológicas,^(^ 23 ^)^ suas informações são de grande valia para uma estimativa da incidência do MM, além de reforçar a concepção que, de fato, é necessário um melhor monitoramento da incidência do MM pelas autoridades competentes para a disponibilização de um banco de dados fidedignos sobre a incidência do MM no Brasil, o que já realidade nos EUA.^(^ 22 ^)^ Além daquele estudo de cunho epidemiológico,^(^ 23 ^)^ outros esforços científicos brasileiros foram realizados na busca por melhorias no diagnóstico^(^ 25 ^,^ 26 ^)^ e por marcadores de prognóstico do MM.^(^ 27 ^)^

Diante da sua agressividade e da crescente incidência do MM a nível mundial, esse câncer e seu principal agente etiológico, o amianto, têm sido fruto de discussões internacionais, as quais tentam banir o comércio desse mineral em todo o mundo. Entretanto, o amianto no Brasil é regulamentado pela Lei nº 9.055, sendo todas as formas de amianto proibidas, com a exceção da crisotila.^(^ 28 ^)^ Logo, pouco é pesquisado sobre esse câncer e, como consequência, conforme dito anteriormente, há poucos estudos sobre o MM e o perfil das pessoas diagnosticadas com esse câncer no Brasil,^(^ 26 ^,^ 29 ^-^ 33 ^)^ e, portanto, estudos mais detalhados são necessários.

Diante desse cenário, a presente revisão teve como objetivo principal fornecer um panorama de como o MM utiliza o maquinário celular para promover seu crescimento, ou seja, quais genes e vias são ativados ou desativados, visto a importância desse conhecimento para o desenvolvimento de novas terapias e fármacos para o combate desse câncer tão agressivo. Deve-se ressaltar que não objetivamos revisar os papeis do amianto e de outros fatores de exposição ambientais no desenvolvimento do MM. Portanto, nós como autores do presente artigo, tivemos como objetivo principal revisar os principais eventos bioquímicos e genéticos que ocorrem no MM e suas respectivas consequências no processo de transformação para malignidade, na tentativa de fortalecer a literatura científica brasileira, que carece de uma revisão da literatura escrita em nossa língua pátria sobre esse tema.

## Genes e vias bioquímicas envolvidas no MM

O conhecimento dos processos celulares que favorecem ou auxiliam no processo de desenvolvimento do câncer é de extrema importância para a criação de possíveis terapias que visem ativar ou desativar determinadas vias bioquímicas, tendo como efeito principal a supressão do crescimento tumoral. Grupos de pesquisa espalhados pelo mundo estão trabalhando nesse ramo, e, mesmo com poucos grupos envolvidos, grandes avanços ocorreram, auxiliando o tratamento desse câncer tão agressivo. Diante disso, nas próximas linhas, descreveremos de forma sucinta os principais genes que participam de forma chave no desenvolvimento do MM. Todavia, esse não foi o foco do presente estudo. Portanto, para os leitores que desejem uma leitura mais profunda sobre a mecânica dos genes envolvidos no MM. Portanto, para os leitores que desejem uma leitura mais profunda sobre a mecânica dos genes envolvidos no MM, recomendamos a leitura de artigos de revisão publicados recentemente pelo nosso grupo.^(^ 21 ^,^ 34 ^)^

Sabe-se que cada câncer utiliza um determinado ''grupo'' de genes para o seu crescimento; contudo, esse ''grupo'' varia com o tipo e o estágio do câncer. Determinados padrões de ativação e/ou desativação dos genes ocorrem em todos os tipos de câncer, os quais são explorados no desenvolvimento de fármacos e terapias. No caso particular do MM, os genes que possuem seus papeis bem estabelecidos são os seguintes: *p16* *INK4a*, *p14* *ARF*,* NF2* e *BAP1*. Entretanto, apesar dos papeis bem estabelecidos dos genes *TP53 *e* PTEN *em diversos outros tipos de câncer, o papel desses ainda é controverso no MM. A Figura 1 mostra um resumo da função desses genes. 

**Figura 1:** Envolvimento dos genes e suas respectivas proteínas no desenvolvimento do mesotelioma maligno. As proteínas p16INK4a e p14ARF atuam na ativação da via da proteína do retinoblastoma (pRb) e modulação da p53, respectivamente. O NF2 codifica a proteína merlin, a qual atua com um regulador upstream da via bioquímica Hippo. BAP1 codifica a proteína BRCA1 associated protein-1 (BAP1 proteína-1 associada a BRCA), que é responsável na resposta frente a danos ao DNA e também no ciclo celular. PTEN codifica a proteína phosphatase and tensin homolog (PTEN, homólogo de tensina e fosfatase), a qual é uma importante reguladora negativa da via PI3K/Akt. A p53 atua de forma chave no controle da apoptose e senescência celular.

### Genes p16INK4a e p14ARF

Localizados no cromossomo 9p21, os genes *p16* *INK4a* e* p14* *ARF* são conhecidos por serem importantes supressores de crescimento tumoral e codificam duas proteínas distintas, denominadas de p16^INK4a^ e p14^ARF^. A p16^INK4a^ é uma inibidora da quinase dependente de ciclina, a qual atua na hiperfosforilação da proteína do retinoblastoma, resultando na inativação da mesma e consequente falha na parada do ciclo celular. Ao contrário, a p14^ARF^ inibe a degradação da proteína p53 através da interação com a proteína *mouse double minute 2* (MDM2, murino duplo minuto 2).^(^ 35 ^)^ Diante disso, a perda desses genes vicinais gera grandes consequências no controle do ciclo celular, e, por isso, é possível inferir o motivo pelo qual esses genes são os que mais comumente apresentam mutações no MM.

De fato, a literatura demonstra que o *p16* *INK4a* e o *p14* *ARF* estão deletados em 80-90% dos casos de MM.^(^ 36 ^,^ 37 ^)^ Do ponto de visto histológico, o MM do tipo epitelial apresenta aproximadamente 70% de alteração nesses genes, enquanto os demais (bifásico e sarcomatoide) apresentam taxas próximas a 100%.^(^ 38 ^)^ A literatura demonstra que ambos os genes, *p16* *INK4a* e *p14* *ARF*, e suas respectivas proteínas desempenham papeis importantes no controle do ciclo celular e que suas respectivas inativações são as mais frequentemente implicadas durante o processo de transformação maligna do MM. 

### Gene NF2

Presente no cromossomo 22q12, o gene *NF2* codifica uma proteína com uma sequência de 595 aminoácidos, denominada merlin, a qual atua de forma importante na regulação *upstream* da cascata da via bioquímica Hippo, a qual será explicada posteriormente. Em meados da década de 1990 foi inicialmente relatada a inativação desse gene em cerca de 40% dos MM.^(^ 39 ^)^ De fato, estudos posteriores demonstraram a importância da inativação desse gene no MM.^(^ 40 ^)^ De forma interessante, recentemente foi relatada a ausência de mutações no *NF2* no câncer de pulmão de não pequenas células, enquanto foram encontradas mutações em 38% dos casos de MPM, demonstrando assim uma possível abordagem de distinção e diagnóstico entre ambos os tipos de câncer.^(^ 41 ^)^ Diante disso, mutações e/ou alterações no *NF2 *são de importante valor para o desenvolvimento do MM, sendo considerada, até o presente momento, a segunda alteração mais frequente nesse câncer. 

### Gene BAP1

O gene *BAP1* é um supressor de tumor localizado no cromossomo 3p21.3 e codifica a proteína *BRCA1 associated protein-1* (BAP1 proteína-1 associada a BRCA), a qual possui um importante papel na via ubiquitina-proteassoma na desubiquitinação das histonas, na regulação da progressão do ciclo celular, na modulação da cromatina, na transcrição gênica e no reparo de DNA.^(^ 42 ^)^

Recentemente, mutações germinativas no *BAP1* foram detectadas em famílias com elevados índices de incidência de MM, caracterizando assim uma síndrome que predispõe ao desenvolvimento de MM, melanoma da úvea e possivelmente outros tipos de câncer.^(^ 19 ^,^ 42 ^,^ 43 ^)^ Além dessas mutações germinativas, mutações somáticas também foram identificadas em aproximadamente 20% dos MM.^(44,45) ^Estudos^(^ 19 ^,^ 42 ^,^ 43 ^)^ sobre o efeito da mutação germinativa no desenvolvimento do câncer trouxeram um grande avanço, pois frequentemente o câncer é associado ao efeito da mutação somática relacionada ou não a fatores externos, tais como amianto, cigarro e radiação, entre outros. Diante disso, é de suma importância melhor compreender quais genes e por quais mecanismos as mutações germinativas atuam no desenvolvimento do MM, pois pessoas que possuem tais suscetibilidades genéticas devem evitar ao máximo a exposição aos fatores condicionantes desse câncer. Para tanto, são necessárias técnicas capazes de detectar essas mutações na população de forma acessível e reprodutível, pois até o momento tal monitoramento é realizado em pequena escala e em estudos científicos. O diagnóstico precoce em indivíduos suspeitos com síndrome *BAP1* é fundamental para prevenir o surgimento de doenças associadas com as mutações desse gene. Dessa forma, torna-se necessária uma abordagem multidisciplinar que envolva médicos de família, patologistas e geneticistas, a fim de detectar, acompanhar, aconselhar e tratar indivíduos e famílias acometidos por essa síndrome. São necessários o conhecimento e o treinamento dos profissionais de saúde, em especial os médicos, em relação aos sinais clínicos dessa síndrome, a qual, se não diagnosticada precocemente, pode trazer danos catastróficos ao paciente.^(^ 42 ^)^

### Gene TP53

Conhecida como a guardiã do DNA, a proteína p53 é codificada pelo gene *TP53*. A p53 atua em diversas funções celulares de suma importância para um controle celular orquestrado. Sabe-se também que o *TP53* apresenta mutações em aproximadamente 50% em todos os tipos de câncer, e, na maioria dos demais casos, o *TP53 *está inativado devido a mutações em outros genes e/ou a proteínas virais.^(^ 46 ^,^ 47 ^)^

Do ponto de vista mecanístico, vários grupos de pesquisa têm focado em melhor compreender os diversos mecanismos de controle das funções celulares da p53. Na década de 1990, as primeiras pistas foram descobertas sobre a ação da p53 na mediação da apoptose.^(^ 48 ^)^ Recentemente, um novo mecanismo de ação da p53 foi identificado, e acredita-se ser este um dos principais mecanismos utilizados para o combate do processo de transformação maligna, a senescência celular induzida.^(49) ^Todavia, o papel da p53 em MM ainda não está bem definido, devido ao fato intrigante de que o MM, na grande maioria dos casos, não neutraliza a ação dessa proteína de forma direta, ou seja, através de mutações do *TP53*. Diante disso, estudos demonstram que o *TP53* está presente em seu estado natural, ou seja, sem mutações.^(^ 50 ^,^ 51 ^)^ Uma ressalva deve ser feita, pois o fato de um gene não apresentar mutação não necessariamente implica que o mesmo está funcionante, pois há diversos mecanismos de regulação gênica, como metilação de DNA (regulação epigenética) e RNA de interferência (regulação pós-transcricional), que podem conferir inativação gênica.^(^ 52 ^)^

Também é possível especular que mutações em outros genes, como as citadas acima, são capazes de levar a transformação maligna no MM, havendo assim reduzida pressão seletiva para a inativação do *TP53*. Até mesmo é plausível supor que o desenvolvimento maligno do MM acontece por vias independentes da ação da p53. Assim, ainda há muito a se investigar e compreender os mecanismos que levam a manutenção do estado selvagem do gene *TP53 *no MM. Portanto, até o presente momento, mutações e/ou alterações no *TP53* não parecem ser fundamentais para o desenvolvimento e a progressão do MM.^(^ 53 ^,^ 54 ^)^

### Gene PTEN

Inicialmente descoberto em 1997 por dois grupos independentes,^(^ 55 ^,^ 56 ^)^ o gene *PTEN* é conhecido por ter uma frequente deleção no cromossomo 10. Mutações monoalélicas são comuns em diversos tipos de câncer; entretanto, mutações homozigóticas do *PTEN* são frequentemente encontradas em cânceres avançados, tais como no câncer de endométrio e glioblastoma.^(^ 57 ^)^ De forma interessante, o *PTEN* é fortemente regulado por diversos processos de regulação gênica em vários níveis, tais como RNA de interferência, metilação, acetilação, oxidação e ubiquitinação. Portanto, é de vital importância ter em mente que a análise do status do gene, ou seja, seus níveis de mutação, é de fato importante, mas não deve ser utilizada exclusivamente como parâmetro de predição de ativação e função gênica. A análise dos níveis de expressão proteica também é necessária para conferir a suscetibilidade para o desenvolvimento do câncer, como é o caso intrigante do *PTEN*.^(^ 52 ^,^ 58 ^)^

A atividade da proteína *phosphatase and tensin homolog* (PTEN, homólogo de tensina e fosfatase) decorre da capacidade de antagonizar a via de sinalização da via do *phosphatidylinositol 3-kinase* (PI3K, fosfatidilinositol-3-quinase) mediante a desfosforilação do *phosphatidylinositol-3,4,5-trisphosphate* (PIP3, fosfatidilinositol trifosfato) a *phosphatidylinositol 4,5-bisphosphate* (PIP2, fosfatidilinositol bifosfato). Sabe-se que o PIP3 é um segundo mensageiro responsável pela ativação de Akt (ou PKB), a qual, por sua vez, envia sinais necessários para o crescimento, a sobrevivência e a proliferação celular. De fato, vários tipos de câncer apresentam superexpressão nessa via bioquímica, resultando assim em crescimento celular descontrolado. A perda da atividade da PTEN resulta em acumulação de PIP3 e consequente superativação da Akt, sendo então comumente utilizada em processos malignos.^(^ 59 ^)^ Além das atividades citoplasmáticas, a PTEN possui atividades nucleares importantes para o controle do ciclo celular e estabilidade genômica.^(^ 59 ^,^ 60 ^)^

De forma interessante, a PTEN é capaz de regular os níveis de p53 de forma independente de sua atividade como fosfatase, através da manutenção da acetilação da p53 (Figura 2).^(^ 61 ^)^ Ademais, a PTEN inibe a fosforilação do MDM2, a qual é necessária para a migração nuclear e a consequente degradação da p53. Logo, a PTEN é capaz de proteger a p53 da degradação do MDM2.^(^ 62 ^,^ 63 ^)^

**Figura 2:** A proteína phosphatase and tensin homolog (PTEN, homólogo de tensina e fosfatase) protege a p53 da degradação mediante a inibição da migração da proteína murino duplo minuto 2 (MDM2) para o núcleo. Existe um mecanismo cross-talk entre PTEN e p53. Quando ocorre a completa perda de PTEN associada a um TP53 selvagem resulta em um mecanismo de senescência denominado PTEN loss induced cellular senescence (PICS, senescência celular induzido por perda do PTEN), o qual é um importante mecanismo de oposição ao crescimento maligno. A perda de PTEN em um contexto celular com um TP53 mutante tem como consequência um câncer mais agressivo de próstata e leucemia, sendo ambos os mecanismos até o momento demonstrados em animais.

Recentemente, um novo mecanismo foi identificado em câncer de próstata, no qual a deleção completa de *PTEN *associada com *TP53* sem mutação levou de forma surpreendente a uma forte senescência celular induzida, resultando na parada do crescimento celular maligno. Todavia, a deleção completa de *PTEN* associada com *TP53 *com mutação foi associada a uma forma mais grave de câncer de próstata. Diante disso, é plausível especular o motivo pelo qual a deleção completa (homozigótica) de *PTEN* é restrita aos cânceres avançados.^(^ 52 ^)^

Em MM, o papel desse gene ainda é controverso, pois de fato sabe-se que a via bioquímica da PI3K/Akt apresenta superexpressão; no entanto, ainda é amplamente debatido se essa superexpressão é decorrente da ausência ou da inativação do *PTEN*, bem como o seu papel no desenvolvimento do MM.^(^ 64 ^-^ 67 ^)^

### Metilação de DNA e microRNA

Estudos recentes apontam os parâmetros de metilação de DNA e microRNA como fortes fatores que auxiliam no desenvolvimento do câncer e como ferramentas a serem exploradas no tratamento e diagnóstico do mesmo. Em MM, a análise epigenética do perfil de metilação de vários genes foi capaz de distinguir entre tecidos normais e malignos, fato que é de grande importância devido à dificuldade da distinção entre os mesmos,^(^ 68 ^)^ além de poder se tornar uma ferramenta formidável para o diagnóstico de MM.^(^ 69 ^)^

Estudos de microRNA têm demonstrado resultados interessantes. Os microRNAs são capazes de regular e modular a expressão gênica, sendo a expressão desses bastante alterada no câncer.^(^ 70 ^)^ De fato, foram demonstradas diferenças entre a expressão dos microRNAs entre tecidos normais e malignos, além de perfis de expressão específicos de microRNAs em cada tipo histológico de MM.^(^ 71 ^)^ Ademais, intensas pesquisas têm sido desenvolvidas, sendo proposto o uso desses em MM como ferramenta de diagnóstico,^(^ 71 ^)^ marcadores de prognóstico^(^ 72 ^)^ e um possível tratamento para o MM.^(^ 73 ^)^ Portanto, o futuro parece promissor para esses dois parâmetros e para seus benefícios em potencial no diagnóstico e tratamento do MM.

## Vias bioquímicas envolvidas no desenvolvimento do MM

Descrevemos brevemente as vias bioquímicas mais utilizadas no MM durante o processo de transformação maligna, as quais estão resumidas na Figura 3. Conforme dito anteriormente, aos leitores que desejarem uma leitura mais profunda do ponto de vista mecanístico das vias envolvidas no MM recomendamos a leitura de artigos de revisão publicados recentemente pelo nosso grupo.^(^ 21 ^,^ 34 ^)^

**Figura 3:** Vias bioquímicas mais comumente alteradas no mesotelioma maligno. Em a, os receptores tirosina quinase são frequentemente ativados no mesotelioma maligno, gerando assim um aumento das vias bioquímicas Ras e PI3K. A via Ras ativa a Raf, a qual causa fosforilação de mitogen-activated protein kinase kinase (MEK, quinase de proteína quinase ativada por mitógeno) que, por sua vez, causa a fosforilação de mitogen-activated protein kinase (MAPK, proteína quinase ativada por mitógeno), que migra para o núcleo, regulando assim a expressão gênica. Em b, a via Wnt controla diversos processos celulares, sendo que, na presença de um ligante Wnt, um complexo que envolve as proteínas disheveled homolog (Dvl), Axin, frizzled (Fz) e low-density lipoprotein receptor-related (LRP5/6, receptor relacionado a lipoproteína de baixa densidade), leva a inibição da fosforilação da ß-catenina e de sua degradação. Dessa forma, a ß-catenina migra para o núcleo, onde interage com o complexo Tcf/Lef, levando assim a ativação de genes responsíveis. Em c, a proteína merlin é codificada pelo NF2 e inibe a via PI3K e mTOR, atuando como um regulador upstream da via Hippo. Através de um estímulo, uma cascata bioquímica é iniciada, onde macrophage stimulating 1/2 (MST1/2, estimuladora de macrófago 1/2) causa a fosforilação de salvador homolog 1 (SAV1, homólogo salvador), large tumor suppressor 1/2 (LATS1/2, supressor de tumor grande) e Mps one binder kinase 1 (MOB1, um ligante Mps quinase 1). O complexo MST1/2 e SAV1 causam a fosforilação de LATS1/2; já o complexo formado por LATS1/2 e MOB1 interage diretamente e causa a fosforilação de YAP/TAZ, sendo que esse último, quando fosforilado, leva a degradação proteica, enquanto em sua forma desfosforilada, entra no núcleo e se liga aos fatores de transcrição TEAD1-4 a fim de regular genes envolvidos na proliferação e morte celular. Em d, a via PI3K/Akt/mTOR é ativada mediante a conversão de phosphatidylinositol-3,4,5-trisphosphate (PIP3, fosfatidilinositol trifosfato) em phosphatidylinositol 4,5-bisphosphate (PIP2, fosfatidilinositol bifosfato), sendo que PTEN atua como um antagonista dessa ativação. PIP3, pyruvate dehydrogenase kinase, isozyme 1 (PDK1, quinase piruvato desidrogenase, isoenzima 1) e mammalian target of rapamycin (mTOR, alvo da rapamicina em mamíferos) causam a fosforilação da proteína quinase B (Akt), a qual, então ativada, atua em processos-chave na proliferação, sobrevivência e motilidade celular.

### Receptores tirosina quinase

Os receptores tirosina quinase compreendem uma grande família de receptores que regulam o ciclo celular e estão frequentemente ativados no MM.^(^ 74 ^)^ Dentre esses, o EGFR foi detectado em 44% dos MPMs.^(^ 75 ^)^ O VEGF também é expresso por esse câncer e está relacionado com uma redução na sobrevida do paciente.^(^ 76 ^)^ Além desses, o *insulin-like growth factor* (fator de crescimento semelhante à insulina) e seu respectivo receptor estão ativos nesse câncer.^(^ 77 ^)^

A ativação desses receptores tem como consequência a ativação de cascatas bioquímicas que levam à transdução de sinais anormais de crescimento celular, principalmente pelas vias das Ras/MAPK^(^ 78 ^,^ 79 ^)^ e PI3K/Akt^(^ 80 ^)^ em MM.

### PI3K/Akt/mTOR

A via do PI3K regula diversos processos vitais para a célula, como a sobrevivência, o metabolismo e a proliferação, entre outros. O PIP3 é um dos principais produtos dessa via, o qual atua como um segundo mensageiro essencial para a translocação da Akt para a membrana, onde essa é fosforilada. A proteína Akt em sua forma fosforilada é responsável pelo envio de sinais bioquímicos responsáveis pela proliferação celular e resistência à apoptose.^(^ 64 ^)^ O gene *PIK3CA* codifica a unidade catalítica p110α, a qual também é conhecida por sua habilidade de ativar a via do PI3K mediante a conversão de PIP2 em PIP3.^(^ 81 ^)^ Sabe-se que a Akt é fosforilada pelo *mammalian target of rapamycin* (mTOR, alvo da rapamicina em mamíferos), sendo que o complexo mTOR possui um importante papel no balanço energético e no crescimento, tornando-se assim um alvo terapêutico de interesse no MM.^(^ 82 ^)^

No MM, essa via apresenta de fato superexpressão,^(^ 64 ^,^ 80 ^,^ 83 ^)^ tornando-se então uma ótima via terapêutica mediante a inibição da atividade de componentes dessa via, como PI3K e mTOR.^(^ 84 ^)^ Entretanto, a aplicabilidade desses fármacos na clínica médica tem se provado frustrante.^(^ 85 ^)^

Recentemente, em um elegante estudo,^(^ 86 ^)^ foi demonstrado que a ativação do *colony-stimulating factor 1 receptor* (CSF1R, fator receptor estimulador de colônia de macrófagos) é capaz de gerar clonogenicidade e resistência em células mesoteliais não transformadas. Ademais, foi demonstrado que em culturas primárias e em linhagens celulares de MPM existem subpopulações de células que expressam CSF1R, o qual é responsável pela resistência ao pemetrexede mediante a sinalização pela Akt e β-catenina. Outro achado interessante daquele estudo é que essa subpopulação de células representa menos do que 10% do número total de células em cultura, sendo essa pequena fração responsável pela resistência ao pemetrexede em linhagens celulares e culturas primárias, demonstrando assim um importante papel do CSF1R na sobrevivência de células que não expressam tal fator.^(^ 86 ^)^ Portanto, devido à grande influência do CSF1R na sobrevivência celular, é de fato atrativa e promissora a inibição farmacológica em humanos desse fator, visto que há uma maior expressão do CSF1R no MM em comparação ao tecido normal, a fim de vencer a grande resistência à quimioterapia observada no MM e aumentar assim o tão limitado arsenal terapêutico contra esse câncer.^(^ 86 ^)^

### Ras/MAPK

A via Ras/MAPK compreende diversos componentes, como receptores de superfície e fatores de transcrição, os quais regulam a expressão gênica. Essa via é uma das que apresentam mais frequentemente desregulação no câncer em linhas gerais e controla processos vitais da célula, como a proliferação, o crescimento e a senescência, além de regular a apoptose através da interação com várias proteínas da família *B-cell lymphoma* (Bcl, linfoma de células B).^(^ 87 ^)^ Os principais constituintes dessa via são Ras, Raf, MEK e MAPK, os quais estão suscetíveis a mutações e/ou alterações e consequentemente favorecem o processo de transformação maligna. De fato, devido à importância dessa via, vários fármacos foram e estão em desenvolvimento e alguns desses estão em fase de ensaio clínico.^(^ 88 ^)^

Estudos demonstram que há um aumento da expressão de MAPK no MM em comparação ao tecido pulmonar normal,^(^ 89 ^)^ além de ativação prolongada da MAPK após a exposição ao amianto,^(^ 78 ^)^ demonstrando assim que essa via é de interesse no crescimento do MM e que sua inibição pode trazer resultados interessantes no tratamento do MM.

### Família de proteínas Bcl e apoptose

Responsáveis pelo controle da apoptose, a família de proteínas Bcl é dividida em duas classes principais: proapoptóticas e antiapoptóticas.^(^ 90 ^)^ O MM, assim como os demais tipos de câncer, apresenta resistência a apoptose, dificultando assim a destruição das células malignas pela quimioterapia tradicional.^(^ 91 ^)^ De fato, o MM expressa diversos membros antiapoptóticos,^(^ 92 ^)^ o que reduz a eficiência da quimioterapia tradicional. Fármacos inibidores dos membros da Bcl foram desenvolvidos e demonstraram resultados interessantes, mas sua aplicação clínica até o presente momento é ainda desconhecida.^(^ 93 ^)^

### Hippo

A via Hippo controla a proliferação, o crescimento e a morte celular através de uma complexa cascata de eventos bioquímicos que resulta em regulação gênica.^(^ 94 ^)^ No MM, essa via foi identificada mediante a perda da expressão do gene *LATS2*, além da expressão do oncogene *YAP* e de um regulador *upstream *dessa via, como merlin, que é codificada pelo gene *NF2*.^(^ 95 ^)^ Diante disso, essa via bioquímica é utilizada pelo processo de transformação maligna do MM. Todavia, mais estudos são necessários para se compreender por quais mecanismos o MM utiliza essa via para seu benefício. 

### Wnt

A via Wnt regula processos celulares de importância, como a proliferação, a polaridade e a morte celular durante o desenvolvimento embrionário e no processo de progressão tumoral. ^(^ 96 ^)^ Em linhas gerais, a ativação dessa via ocorre por duas formas: alteração do processo de transcrição, denominada de ativação canônica, ou mediante ativação de processos não transcricionais, denominada de ativação não canônica.

Sabe-se também que a β-catenina é o principal agente transcricional dessa via, atuando no núcleo e formando um complexo molecular que leva a ativação de genes específicos.^(^ 97 ^)^ No MM, essa via foi demonstrada estar alterada,^(^ 98 ^,^ 99 ^)^ bem como foi implicada na diminuição da sobrevivência de pacientes.^(^ 100 ^)^

## Relevância dos processos de sinalização alterados no câncer

Diante do que foi dito acima, podemos dizer com clareza que compreender o complexo e enigmático processo bioquímico e molecular que ocorre durante o processo de desenvolvimento maligno é de suma importância para o desenvolvimento de novos fármacos e terapias. A busca pelo conhecimento de como a célula maligna consegue subverter a maquinaria celular e todos os sistemas de controle do ciclo celular tem se tornado exaustiva; entretanto, através dessa, benefícios têm sido alcançados, os quais resultam em novas terapias e fármacos que aumentam as chances de sobrevivência de um paciente acometido pelo câncer.

### Individualização: o futuro do tratamento do câncer

De fato, o conhecimento profundo de como o câncer utiliza a maquinaria celular para dirigir seu crescimento é de suma importância, pois cada câncer, como dito anteriormente, utiliza genes e vias distintas, gerando assim ''padrões'' de ativação e inativação.^(^ 101 ^)^ Esses ''padrões'' podem nos fornecer pistas importantes para o desenvolvimento de terapias e fármacos específicos para o câncer.

Conhecer com profundidade os processos moleculares que ocorrem em um determinado paciente é o ramo da medicina personalizada, a qual busca tratar cada doença, em nosso caso o câncer, de forma individual devido à grande variabilidade dos processos fisiológicos, trazendo assim uma possibilidade de melhora no tratamento e prognóstico. Todavia, tal abordagem ainda se encontra em seus primeiros passos, mas acreditamos que no futuro tal abordagem possa ser utilizada na clínica.^(^ 102 ^)^

Já é conhecida, e em alguns casos recomendada, a análise do perfil de determinadas enzimas em pacientes que recebem certos medicamentos que são metabolizados por enzimas específicas. Tais enzimas sofrem variações de diversos fatores genéticos, os quais culminam com alterações no perfil farmacocinético e farmacodinâmico dos fármacos, resultando assim em falha terapêutica e/ou aumento dos efeitos adversos.^(^ 103 ^)^

O mesmo se aplica ao câncer, pois o câncer tem sido tratado como uma doença de massa, enquanto, na verdade, deveria ser tratado de forma personalizada. Logo, conhecer os receptores e as vias que estão sendo expressos pelo o câncer em particular é fundamental durante a escolha de fármacos específicos para aquele cenário celular e, portanto, através da análise desses fatores, ou seja, quais vias e receptores estão sendo expressos ou não, podemos então propor a utilização de fármacos específicos para que atuem nos alvos utilizados pelo câncer. Dessa forma, tal abordagem traz um grande benefício durante o tratamento, além de aumentar as chances de sobrevivência do paciente. Portanto, com os grandes avanços tecnológicos e no conhecimento referente ao processo de transformação maligna, acreditamos cada dia mais que se aproxima o tempo de uma medicina personalizada, baseada e norteada nos processos celulares desregulados que ocorrem no paciente, propondo e utilizando assim, ferramentas terapêuticas específicas e obviamente aumentando as chances de combate do câncer.

## Considerações finais e perspectivas

Tendo o que foi dito acima em mente, o objetivo motivador principal do presente estudo foi a carência de revisões da literatura referente aos processos genéticos e bioquímicos utilizados no MM publicados na língua portuguesa. De fato, esse câncer não é amplamente pesquisado no Brasil, havendo poucos grupos nesse país que se dedicam ao estudo epidemiológico do MM. Associado a isso, há poucas fontes de referência sobre a incidência e o perfil desse câncer na população brasileira, além da ausência de um número fidedigno quanto a incidência e prevalência de casos de MM no Brasil. 

Como citado anteriormente, nosso grupo tem se empenhado em melhor compreender os processos de sinalização celular que contribuem para o processo de tumorigênese do MM. Contudo, a ausência de trabalhos escritos em português disponíveis para todos os brasileiros sobre esse tema nos incentivou a escrever o presente estudo, no qual tentamos trazer uma visão panorâmica dos eventos genéticos e bioquímicos mais comumente utilizados durante o processo de transformação maligna do MM. Entretanto, não foi nosso objetivo, como citado anteriormente, revisar profundamente essas vias, mas sim fornecer uma visão geral dos processos de sinalização celulares que ocorrem no MM. Diante disso, podemos concluir que o MM é um câncer extremamente agressivo, com elevado período de latência e com baixíssimas taxas de sobrevivência. No mundo inteiro, grande esforços têm sido executados a fim de melhor compreender o processo de tumorigênese do MM para propor e desenvolver alternativas no combate e tratamento desse câncer tão agressivo. Além disso, deve ser ressaltado que não foi do escopo do presente estudo analisar os fatores associados com o MM, tais como amianto, erionita e vírus símio 40.

Demonstramos que o MM apresenta vários genes que comumente apresentam-se com mutações, tais como *p16* *INK4a*,* p14* *ARF*,* NF2 *e* BAP1*, cujas mutações apresentam-se comumente de forma somática. Vimos também que mutações germinativas do *BAP1 *foram recentemente identificadas, conferindo suscetibilidade ao desenvolvimento de MM e a outros tipos de câncer. Por fim, vimos também que o *TP53 *e* PTEN*, apesar de serem genes de grande importância em outros tipos de câncer, carecem de estudos mais aprofundados quanto a seus respectivos papeis no MM. Além desses genes, as vias PI3K/Akt/mTOR, Ras/MAPK, Bcl, Hippo e Wnt e seus respectivos membros apresentam-se como as mais alteradas no MM, sendo que diversos esforços e estudos têm focado na modulação dessas vias para levar de forma segura e eficaz a uma redução no processo de crescimento maligno do MM, inicialmente a partir de estudos in vitro, seguido de estudos em animais e finalmente em ensaios clínicos. 

É claro que mais estudos são necessários a fim de melhor conhecer e caracterizar as vias e genes atuantes no processo de tumorigênese do MM, os quais infelizmente não têm sido alvo dos esforços científicos brasileiros. Tais esforços são necessários, bem como são urgentes um maior número de estudos epidemiológicos que visem analisar o perfil da incidência do MM na população brasileira, traçando assim um perfil realista e fidedigno desse câncer em nosso país.
