# Supplementary material for: ERRATUM
Source: J Bras Pneumol. 2014 Sep-Oct;40(5):588. doi: 10.1590/S1806-37132014000500019 (PMC4263344; doi:10.1590/S1806-37132014000500019)
Supplement: Translation

# Translation: ERRATA

**Artigo:** Panorama dos processos bioquímicos e genéticos presentes no mesotelioma maligno 

**Publicação:** Jornal Brasileiro de Pneumologia. 2014;40(4):429-42

**DOI:**10.1590/S1806-37132014000400012 

Na página 429 da publicação original, na resumo, na 3° linha, onde se lê "O desenvolvimento do MM é fortemente correlacionado com a exposição ao amianto e erionita, assim como ao vírus símio 40." leia-se "O desenvolvimento desse câncer é correlacionado com a exposição ao amianto e a outros fatores, como a erionita e o vírus SV40".
